# Delayed-type hypersensitivity in classic Kaposi sarcoma patients and controls

**DOI:** 10.1038/sj.bjc.6606088

**Published:** 2011-01-18

**Authors:** R M Valenti, E Amodio, J-M Nam, L Preiss, B I Graubard, N Romano, J J Goedert

**Affiliations:** 1 Section of Hygiene, Department of Sciences for Health Promotion ‘G D’Alessandro', Università degli Studi di Palermo, Palermo 90127, Italy; 2 Division of Cancer Epidemiology and Genetics, National Cancer Institute, 6120 Executive Boulevard, Room 7068, Rockville, MD 20852, USA; 3 RTI International, Rockville, MD 20852, USA

**Keywords:** delayed-type hypersensitivity, Kaposi sarcoma, herpesviruses, human immunodeficiency virus (HIV), transplantation, Italy

## Abstract

**Background:**

Immune perturbation likely affects the development of Kaposi sarcoma (KS) among people infected with the KS-associated herpesvirus (KSHV). We tested whether KSHV-seropositive individuals or cases of classic KS (cKS), which typically originates in the leg, had differing delayed-type hypersensitivity (DTH) in the forearm or leg.

**Methods:**

Mantoux DTH with three antigens (Candida, tetanus, PPD) was performed on the forearm and leg of 15 cKS cases, 14 KSHV-positives without KS, and 15 KSHV-negative controls. The diameters of induration responses were compared by group and body site.

**Results:**

Leg DTH was greater than forearm DTH among controls (mean difference 5.6 mm, *P*=0.0004), whereas this was not observed in cKS cases (−2.2 mm, *P*=0.32) or KSHV-positives (0.5 mm, *P*=0.56). Leg-minus-forearm DTH difference was greater in controls compared with cKS cases (*P*=0.004) and KSHV-positives (*P*=0.002). Leg-plus-forearm DTH was similar in controls (mean 28.2 mm) and cKS cases (24.5 mm, *P*=0.60), but it was reduced in KSHV-positives (11.8 mm, *P*=0.02), particularly in the leg (*P*=0.004) and marginally in the forearm (*P*=0.07).

**Conclusion:**

KS cases had weaker DTH only in the leg, whereas both body sites appeared weaker in KSHV-positives without KS. Both systemic and regional immune alterations may influence the development of this malignancy.

Kaposi sarcoma (KS) is caused by infection with the KS-associated herpesvirus (KSHV, also known as human herpesvirus 8), but only a fraction of infected people develop the disease. The risk of KS development is markedly increased for people with a severe perturbation in immunity, especially HIV/AIDS. In contrast, the risk for classic KS (cKS) is approximately 100-fold lower than for AIDS KS (Vitale *et al*, 2001; Engels *et al*, 2003), and the immune defect underlying cKS is unknown.

Thirty years ago, delayed-type hypersensitivity (DTH) responses to dinitrochlorobenzene (DNCB) were noted to be absent in 16 African, non-AIDS KS patients who had very advanced disease but not in 30 who had limited disease (Master *et al*, 1970). Subsequently, two cases of cKS (Ruocco *et al*, 1984; Ruocco and Satriano, 1986) and a small series of African, non-AIDS KS cases (Ziegler and Katongole-Mbidde, 1996) were reported to have seriously deficient DTH in the leg, where the disease typically originates. This study tested two hypotheses – first that DTH was generally low in cKS patients, and then that DTH was specifically deficient in the organ at highest risk – the skin of the leg.

## Materials and methods

### Research participants and KSHV serology

From the 2002–2006 population-based cKS case–control study (Anderson *et al*, 2008), which ascertained cKS cases and randomly sampled controls from the entire island of Sicily, participants for this study were recruited from those who were alive and who lived not more than 1-h driving time from the clinic in Palermo. All such cKS cases and KSHV-seropositives were recruited, as were a random sample of the larger population of KSHV-seronegative controls. During 2009, they were contacted by telephone and given a pair of clinic appointments for intradermal injection of the antigens and measurement of DTH responses 48 h later. As reported in detail (Anderson *et al*, 2008), seropositive subjects had antibodies detected by immunofluorescence assay (IFA) against the KSHV latency-associated nuclear antigen (LANA) or by enzyme immunoassay (EIA) against the KSHV K8.1 antigen; seronegative subjects were non-reactive against KSHV LANA and lytic antigens by IFA and against KSHV K8.1 and ORF73 antigens by EIA. Subjects with indeterminate KSHV serology (Anderson *et al*, 2008) were excluded from this study. The study was approved by institutional review boards at the University of Palermo and at the National Cancer Institute in the USA.

### DTH and data collection

Following informed consent and an examination for cKS lesions, one dermatologist identified disease-free areas on the volar surface of the forearm and the medial surface of the thigh. Using the conventional Mantoux method, six wheals were raised by intradermal injection of 100 *μ*l of each antigen solution (tetanus toxoid (Imovax Tetano, Sanofi Pasteur MSD S.p.a., Rome, Italy); PPD (five tuberculin units); and *C. albicans* (Candin; Allermed Laboratories Inc., San Diego, CA, USA)). Each participant was observed for 30 min for possible acute reactions, of which there were none.

Approximately 48 h after administration, the same dermatologist examined each injection site on the forearm and leg. The perpendicular diameters of palpable induration were determined using the ball-point pen method (Sokal, 1975), measured with calipers, and tabulated. Induration with a minimum diameter ⩾5 mm was categorised as positive, else negative. Cross-sectional area (the product of the perpendicular diameters) was calculated for indurations with minimum diameter ⩾2 mm.

Total lymphocyte counts and CD4 and CD8 T-cell subsets were determined by the HIV/AIDS clinical monitoring laboratory using standard cytometric methods.

### Analysis plan and methods

We compared three groups – cKS cases, KSHV-seropositives, and KSHV-seronegative controls (Anderson *et al*, 2008) – on DTH in the forearm; DTH in the leg; difference in DTH between the forearm and leg (a standardised measure of the leg); and finally sum of DTH in the forearm-plus-leg (a more general measure). The primary measure was the product of the perpendicular diameters of induration areas, which were transformed with the square root function (mean diameter) to better approximate normal distributions. Mean square-root values were transformed back to the original scale for presentation. Within-subject differences in mean induration diameters in the forearm and leg were compared with Student's *t*-test, either paired (within subject) or unpaired (between groups), and with analysis of variance. Categorical data (positive–negative responses) were compared with appropriate tests (Kruskal and Wallis, 1952; Armitage, 1971), but these are not presented because the associations were very similar to those with continuous (mean diameter) responses. Differences in lymphocyte and T-cell subsets were tested with one-way analysis of variance. Mean and standard distribution (s.d.) of induration diameter and cell counts by number of KS lesions in the cases are presented. Two-sided *P*-values ⩽0.05 were deemed to be statistically significant.

## Results

We evaluated 44 participants, of whom 9 were female. The three groups did not differ with respect to age (*P*=0.48, mean 66 years). They included 35 residents of the province of Palermo, and 9 residents of the neighbouring provinces of Trapani or Agrigento. As we reported previously (Anderson *et al*, 2008), diabetes was more common in cases (40%) compared with KSHV-seropositives (zero, *P*=0.02) but not compared with seronegative controls (20%, *P*=0.43). Kaposi sarcoma originated on the foot or leg in all 15 cases except 1; a second case developed metastatic KS on the arm. Nine cases currently had no KS lesions, three had 1–2 lesions, and three had approximately 20 lesions. Delayed-type hypersensitivity was performed on skin sites (volar forearm and medial thigh) that were far from KS lesions. The mean diameter of DTH responses in the forearm and leg of each participant is presented in Supplementary Table S1. Delayed-type hypersensitivity responses did not appear to differ by antibody response against KSHV LANA *vs* K8.1, but the serology was highly concordant. Specifically, nine of the KSHV-seropositives had antibodies against LANA, and all but one had antibodies against K8.1. All cKS cases had antibodies against K8.1, and all but two had antibodies against LANA. As we were interested in cumulative DTH, diameters were summed for each participant, separately in forearm and leg, and then with the two body sites combined.

**Figure 1:**
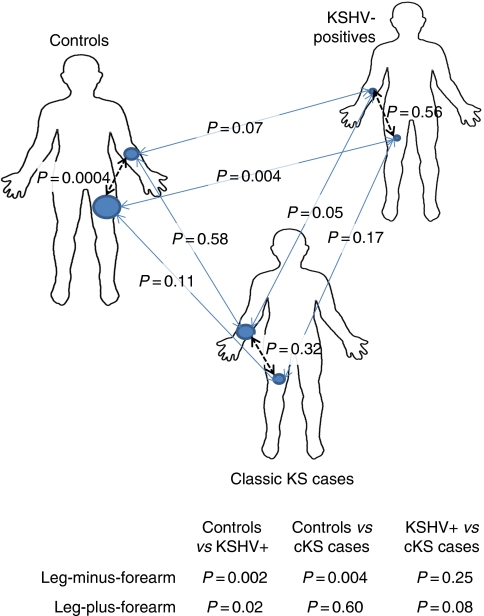
Comparisons of DTH responses within (black dashed lines) and between (blue solid lines) cases of classic KS, KSHV-seropositive people without KS, and KSHV-seronegative controls. Upper panel (body images), blue circles are proportional to the mean responses in the forearm and leg. Lower panel (table), comparisons of differences (leg-minus-forearm) and sums (leg-plus-forearm) in responses.

First, we compared mean induration in the forearm *vs* mean induration in the leg, within each group (Supplementary Table S2). As shown in Figure 1 (black dashed lines in body images), the controls had significantly larger responses in the leg than the forearm (16.9 *vs* 11.3 mm, *P*=0.0004). In contrast, leg and forearm did not differ significantly in the KSHV-positives (*P*=0.56) or cKS cases (*P*=0.32) groups.

Second, we performed pair-wise comparisons of the three groups for size of responses in the forearm, then in the leg (Supplementary Table S2). As shown in Figure 1 (blue solid lines between body images), responses in the forearm were marginally smaller in KSHV-positives (mean 5.7 mm) than in controls (11.3 mm, *P*=0.07) and cKS cases (13.4 mm, *P*=0.05), whereas the latter two groups did not differ from each other (*P*=0.58). In the leg, controls had significantly larger responses in the leg (16.9 mm) compared with KHSV-positives (6.2 mm, *P*=0.004) but not compared with cKS cases (11.2 mm, *P*=0.11). Response sizes in the leg did not differ between cKS cases and KSHV-positives (*P*=0.17).

Within individuals, difference in response sizes by body site was significantly greater in controls (leg-minus-forearm, 5.6 mm) compared with KSHV-positives (0.5 mm, *P*=0.002) and cKS cases (−2.2 mm, *P*=0.004, Figure 1, lower panel). The three groups varied significantly from each other in leg-minus-forearm differences (*P*=0.002), and this remained significant after adjustment for diabetes (*P*=0.003). With Bonferroni adjustment for 15 comparisons (Figure 1), leg-minus-forearm differences were marginally significant (*P*=0.03–0.06).

The sum of responses (leg-plus-forearm) within individuals was significantly lower in KSHV-positives (11.8 mm) compared with controls (28.2 mm, *P*=0.02) and marginally compared with cKS cases (24.5 mm, *P*=0.08), whereas the latter two groups did not differ from each other (Figure 1, lower panel). The three groups varied significantly from each other in leg-plus-forearm DTH (*P*=0.05), and this remained significant after adjustment for diabetes (*P*=0.05).

There were no differences between or across the groups in total lymphocyte counts, CD4 count or percent, CD8 count or percent, or CD4/CD8 ratio (*P*⩾0.20, Supplementary Table S2). The cKS cases with >2 lesions tended to have lower CD8 counts (*N*=3, mean 381±345) than cases with fewer lesions (*N*=3, 573±173) or no lesions (*N*=9, 543±449). Cases with >2 lesions also tended to have smaller induration (7.8±3.2 mm in forearm, 5.3±4.6 mm in leg) compared with cases with fewer lesions (22.0±5.4 mm in forearm, 17.0±10.5 mm in leg) or no lesions (12.3±13.9 mm in forearm, 11.2±10.5 mm in leg).

## Discussion

We sought to identify whether there were differences in DTH, overall or between forearm and leg, which were associated with cKS or with KSHV-seropositivity without KS. We found a large and highly significant difference between the legs and forearms, with stronger responses in the legs of controls. In addition, the KSHV-seropositives had weaker overall responses (arm-plus-leg), including significantly weaker responses in the forearm compared with the cKS cases. These surprising results suggest that cKS cases and seropositives have divergent DTH responses that may relate to the development of KS *vs* remaining KS free.

As our controls have little or no risk of cKS, they represent the basal condition and thereby approximate DTH in this elderly, general population. Compared with these controls, the markedly smaller indurations in the legs of cKS cases support anecdotal reports of fewer and smaller DTH responses in the leg or foot compared with the arm (Ruocco *et al*, 1984; Ruocco and Satriano, 1986; Ziegler and Katongole-Mbidde, 1996). In contrast, DTH responses in the arm against DNCB were observed in all of 30 Ugandan patients who had relatively indolent KS (Master *et al*, 1970). Our cKS cases and controls had equivalent responses in the forearm, supporting the hypothesis that the KS-associated defect in DTH is specifically in the leg, where classic and endemic KS most often originates.

Stronger DTH in the leg of cKS cases compared with seropositives is the opposite of what we anticipated. It suggests that seropositives may be a special and highly informative group. They have survived with KSHV infection without developing KS, generally for several decades. (Whitby *et al*, 2000; Pelser *et al*, 2009) The observation that the KSHV-seropositives had smaller indurations (Figure 1), including the arm, raises the hypothesis that generalised hypo-responsiveness may have reduced their risk for developing KS. This may appear paradoxical, as HIV infection causes a profound deficiency of DTH but an enormous increase in KS risk (Birx *et al*, 1993; Vitale *et al*, 2001; Engels *et al*, 2003). However, people with HIV also have markedly activated immunity. In fact, elevated serum level of *β*_2_-microglobulin or especially neopterin is more predictive of AIDS KS than is low CD4 lymphocyte count (Rabkin *et al*, 1990; Krämer *et al*, 1992), and cKS cases tend to have elevated levels of serum neopterin and *β*_2_-microglobulin (Touloumi *et al*, 1999; Brown *et al*, 2006). Moreover, transplant-associated KS was increased with HLA-mismatched allografts (Mbulaiteye and Engels, 2006), which points more to immune activation than suppression. In this study, lymphocyte and T-cell subset levels were not related to KSHV status, nor did they differ among cKS cases as a whole.

Detection of KSHV viremia is the strongest marker of KS risk yet identified (Whitby *et al*, 1995; Engels *et al*, 2003). Unfortunately, how KSHV-specific immune responses relate to KSHV viremia, to KS incidence, and even to KS progression or regression have not been clearly defined (Flores and Goedert, 2010). It would be of considerable interest to know whether DTH responses are associated with KSHV viremia in KSHV-seropositive adults. On the basis of the strong association between viremia and KS risk, our current data would suggest that viremia should be associated with stronger DTH responses, perhaps more in the arm than the leg.

Our study was significantly limited by its small size, which reflects the rarity of cKS, the relatively low prevalence of people without KS who have unambiguous KSHV-seropositivity (Anderson *et al*, 2008) and the rigors of performing and reading DTH responses in a very elderly population. Nonetheless, except for a paucity of female cKS cases, our groups were well balanced by sex and age, and these participants are likely to be representative of the ambulatory general population (Anderson *et al*, 2008). We did not evaluate DTH responses in the distal leg, where cKS lesions arise most often. We purposely did not test the distal leg or foot in cKS cases to assure that the injections were distant from lesions. However, these comparisons would be of interest, and probably valid, in KSHV-seropositives and in cKS cases who have no lesions near the DTH test site. Finally, with our retrospective case–control design, reverse causality could account for the observed associations, with cKS or KSHV-seropositivity leading to differences in DTH. We observed suggestive differences by number of KS lesions in our cases, which resemble the heterogeneity by stage of KS noted previously in Uganda (Master *et al*, 1970).

In summary, we confirmed that KS cases have diminished DTH responses in the leg, and we also found evidence, albeit weaker, for diminished responses in the arm-plus-leg of elderly KSHV-seropositives who have not developed KS. As we performed several comparisons, these findings might have emerged merely by chance. However, they support the hypothesis that KS, in the absence of HIV infection, is related to intrinsic differences in immune responses, perhaps in the distal extremities even more so than systemically.
