# Supplementary material for: Delayed-type hypersensitivity in classic Kaposi sarcoma patients and controls
Source: Br J Cancer. 2011 Jan 18;104(3):433–6. doi: 10.1038/sj.bjc.6606088 (PMC3049575; doi:10.1038/sj.bjc.6606088)
Supplement: Supplementary Tables S1–S2 [file 6606088x1.doc]

| **Table S1** (two pages). Delayed-type hypersensitivity (DTH) responses against three antigens in the arm and leg. | | | | | | | | | | |  |
| --- | --- | --- | --- | --- | --- | --- | --- | --- | --- | --- | --- |
|  |  |  |  |  | |  |  |  | |  |  |
|  |  |  | **DTH in forearm*** | | | | **DTH in leg*** | | | |  |
| **No.** | **Sex** | **Group†** | **Candida** | **Tetanus** | **PPD** | | **Candida** | **Tetanus** | **PPD** | |  |
| 1 | M | KS case | 0 | 11.5 | 0 | | 0 | 7.9 | 0 | |  |
| 2 | M | KS case | 0 | 0 | 24.0 | | 0 | 0 | 29.0 | |  |
| 3 | M | KS case | 0 | 3.5 | 0 | | 0 | 7.5 | 0 | |  |
| 4 | M | KS case | 12.5 | 19.8 | 11.0 | | 6.3 | 12.6 | 6.9 | |  |
| 5 | M | KS case | 6.0 | 9.9 | 0 | | 9.4 | 3.2 | 0 | |  |
| 6 | M | KS case | 0 | 0 | 5.0 | | 0 | 2.0 | 8.9 | |  |
| 7 | M | KS case | 0 | 6.3 | 0 | | 0 | 7.9 | 0 | |  |
| 8 | M | KS case | 0 | 0 | 0 | | 0 | 0 | 0 | |  |
| 9 | F | KS case | 0 | 0 | 0 | | 0 | 0 | 0 | |  |
| 10 | M | KS case | 0 | 0 | 17.9 | | 4.5 | 0 | 25.5 | |  |
| 11 | M | KS case | 5.5 | 0 | 0 | | 0 | 0 | 0 | |  |
| 12 | M | KS case | 12.2 | 0 | 14.0 | | 9.5 | 0 | 0 | |  |
| 13 | M | KS case | 5.0 | 0 | 0 | | 5.5 | 0 | 0 | |  |
| 14 | M | KS case | 12.5 | 5.0 | 0 | | 7.5 | 0 | 0 | |  |
| 15 | M | KS case | 5.0 | 7.0 | 6.7 | | 0 | 7.0 | 6.5 | |  |
| 16 | M | KSHV+ | 0 | 4.5 | 0 | | 0 | 0 | 0 | |  |
| 17 | F | KSHV+ | 0 | 0 | 0 | | 0 | 0 | 0 | |  |
| 18 | M | KSHV+ | 0 | 13.4 | 5.5 | | 0 | 16.4 | 10.5 | |  |
| 19 | M | KSHV+ | 0 | 0 | 0 | | 0 | 0 | 0 | |  |
| 20 | F | KSHV+ | 0 | 0 | 0 | | 0 | 0 | 0 | |  |
| 21 | M | KSHV+ | 0 | 0 | 0 | | 0 | 0 | 0 | |  |
| 22 | M | KSHV+ | 0 | 0 | 6.5 | | 0 | 0 | 9.0 | |  |
| 23 | M | KSHV+ | 0 | 8.0 | 10.0 | | 0 | 9.0 | 12.0 | |  |
| 24 | F | KSHV+ | 0 | 0 | 0 | | 0 | 0 | 0 | |  |
| 25 | F | KSHV+ | 0 | 0 | 0 | | 0 | 0 | 0 | |  |
| 26 | M | KSHV+ | 3.0 | 0 | 19.0 | | 0 | 0 | 19.0 | |  |
| 27 | M | KSHV+ | 0 | 2.6 | 0 | | 0 | 5.3 | 0 | |  |
| 28 | M | KSHV+ | 0 | 0 | 0 | | 0 | 0 | 0 | |  |
| 29 | M | KSHV+ | 0 | 2.0 | 5.0 | | 0 | 2.0 | 3.0 | |  |
| 30 | M | Control | 0 | 5.5 | 0 | | 10.0 | 6.0 | 0 | |  |
| 31 | M | Control | 0 | 4.0 | 15.4 | | 0 | 2.4 | 18.4 | |  |
| 32 | M | Control | 0 | 0 | 24.0 | | 0 | 8.4 | 21.2 | |  |
| 33 | M | Control | 0 | 0 | 0 | | 0 | 4.5 | 6.5 | |  |
| 34 | F | Control | 0 | 5.5 | 0 | | 0 | 7.5 | 0 | |  |
| 35 | M | Control | 10.6 | 6.5 | 0 | | 17.0 | 8.9 | 0 | |  |
| 36 | M | Control | 0 | 0 | 15.0 | | 0 | 0 | 15.0 | |  |
| 37 | M | Control | 0 | 0 | 0 | | 0 | 5.5 | 0 | |  |
| 38 | M | Control | 10.0 | 0 | 0 | | 11.0 | 0 | 0 | |  |
| 39 | F | Control | 0 | 0 | 17.5 | | 11.4 | 0 | 17.9 | |  |
| 40 | F | Control | 5.9 | 0 | 0 | | 11.4 | 0 | 0 | |  |
| 41 | M | Control | 0 | 12.8 | 0 | | 13.4 | 13.3 | 0 | |  |
| 42 | M | Control | 0 | 0 | 0 | | 0 | 0 | 0 | |  |
| 43 | F | Control | 0 | 8.9 | 15.5 | | 0 | 8.9 | 17.3 | |  |
| 44 | M | Control | 0 | 0 | 12.2 | | 0 | 0 | 17.3 | |  |
|  |  |  |  |  |  | |  |  |  | |  |
| * Mean of cross-sectional diameters, mm. | | | | | | |  |  |  | |  |
| † KSHV+ indicates seropositive individuals without KS. | | | | | | | |  |  | |  |

**Table S2.** Delayed-type hypersensitivity (DTH) and peripheral blood mononuclear cell (PBMC) values, by group.

| DTH and PBMC values* | Controls  (n=15) | KSHV positives  (n=14) | cKS cases  (n=15) |
| --- | --- | --- | --- |
|  |  |  |  |
| Forearm DTH, mean (±SD) | 11.3 (8.3) | 5.7 (8.0) | 13.4 (11.8) |
| Leg DTH, mean (±SD) | 16.9 (9.3) | 6.2 (9.3) | 11.2 (9.8) |
| Leg – Forearm DTH, mean (±SD) | 5.6 (4.6) | 0.5 (3.0) | -2.2 (8.1) |
| Leg + Forearm DTH, mean (±SD) | 28.2 (17.0) | 11.8 (17.1) | 24.5 (20.2) |
| Lymphocyte count, mean (±SD) | 1879 (673) | 2081 (689) | 1993 (646) |
| median | 1720 | 2060 | 1750 |
| CD4 count, mean (±SD) | 761 (331) | 888 (370) | 817 (297) |
| median | 729 | 867 | 743 |
| CD8 count, mean (±SD) | 525 (251.6) | 545 (292) | 517 (376) |
| median | 500 | 475 | 410 |
| CD4%, mean (±SD) | 40.1 (8.2) | 42.4 (9.5) | 41.9 (10.7) |
| median | 40.6 | 39.3 | 38.6 |
| CD8%, mean (±SD) | 28.0 (7.4) | 25.7 (10.6) | 23.8 (12.7) |
| median | 26.2 | 23.6 | 26.4 |
| CD4/CD8 ratio, mean (±SD) | 1.6 (0.8) | 2.0 (1.2) | 3.2 (3.9) |
| median | 1.6 | 1.9 | 1.6 |

* Mean [standard deviation (SD)] of the average DTH diameter, in mm. Lymphocyte, CD8 and CD4 counts as cells/mm^3^.
